# Acute Pneumonia in Childhood*Read before the Hospital Graduates’ Club, November 23, 1893.

**Published:** 1894-03

**Authors:** Thomas S. Southworth

**Affiliations:** New York; Pathologist to the Nursery and Child’s Hospital; Assistant Medical Department, Vanderbilt Clinic; Instructor in Diseases of Children, New York Polyclinic; 19 West Forty-sixth Street


### ACUTE PNEUMONIA IN CHILDHOOD.*

BY THOMAS S. SOUTHWORTH, M. D.,

Pathologist to the Nursery and Child's Hospital; Assistant Medical Department, Vanderbilt Clinic; Instructor in Diseases of Children, New York Polyclinic.

(CONCLUDED FROM FEBRUARY NUMBER.)

2. *Wandering pneumonia* occurs under two clinical forms. With a continuous high temperature, daily examinations may show that additional lobes are involved, one after the other, without revealing the advance upon the temperature chart. Again, the temperature may fall sharply, as if the crisis had been reached, only to rise again promptly with the development of signs of consolidation in another lobe, and this may be repeated several times before the final critical fall takes place.

3. The *gastric cases* are most puzzling even to the careful observer, for the attack begins with severe vomiting, anorexia, diarrhea, and heavily coated tongue, which, together with the referred pain in the epigastrium, lead almost irresistibly to the conclusion that the affection is of gastro-intestinal origin. To add to the difficulty, the appearance of the physical signs is usually delayed, a central pneumonia coming to the surface upon the third or fourth day, and often only appearing a few hours before the development of the crisis, which latter, as if to keep up the rôle which has previously been played, is often accompanied by profuse vomiting. To avoid falling into error in these cases, therefore, we must rely upon our observation of the continuous high temperature which does not fall after suitable treatment directed to the alimentary tract, and the evident pulmonary embarrassment, cough and characteristic respiratory rhythm.

4. In a somewhat similar manner the *cerebral type* of pneumonia is ofttimes misleading, or at the least causes great anxiety during the period of uncertainty. The well-known disproportionate size of the child's brain to its body renders the nervous system especially liable to disturbance in disease, and cerebral symptoms are

*Read before the Hospital Graduates' Club, November 23, 1893.

very common in pneumonia. Although usually not so decided as to obscure the diagnosis, there are other cases where they are so pronounced as to divert the attention from the lungs to the brain, or at least to place us in doubt as to whether there be genuine meningitis, or whether the symptoms are transient and due to the influence of the hyperpyretic blood upon the central system. Early or late convulsions, somnolence, delirium, headache, vomiting, constipation, irregular pupils, strabismus, irregularity of the pulse, retraction of the abdomen, grinding of the teeth, boring of the head into the pillow, may be present in varying combinations. Genuine meningitis, however, is a rare confliction. Irregularities and intermissions of the pulse may exist in either disease, but the characteristic pulse of meningitis is slow, not rapid as in pneumonia.

Nervous symptoms quite frequently occur where there is extensive, and hence more easily recognizable, involvement of the lung, and where the temperature is persistently higher than we find it in meningitis. Steiner* contends that a complicating otitis media may be responsible for many of these symptoms, and cites sixteen cases. From a prognostic standpoint the time of the development of the convulsions is of value, early seizures being of little importance, but late convulsions indicating speedy dissolution within twenty-four hours.†

Returning now to the consideration of the ordinary cases of pneumonia, we find the left lower and right upper lobes most frequently involved. Comparing the two sides, we find the right lung is more often affected than the left, and this preponderance is explained, as in the adult, by the frequent consolidations of the right middle lobe alone.

More than one lobe upon the same or opposite sides may be involved at the same time, or the process may extend to other lobes during the course of the disease. Unlike the adult lung, it is not uncommon for but a portion only of the lobe to show consolidation. The stethoscope high in the axilla will often detect consolidation of the upper lobe which does not reveal itself elsewhere. With superficial breathing the bronchial character of the breath-sounds often

*Jahrb. f. Kinderh.*, n. F. ii.

† Holt. *Medical Record*, April 7, 1888. Cerebral Symptoms of Pneumonia in Children.

disappears. In such cases careful percussion, however, reveals dullness, and if the child be made to cry, the bronchial character returns. Among the complications, bronchitis of the uninvolved lung occasionally supervenes and threatens death by suffocation. Pleurisy is common, and may give rise to pleural friction sounds; it influences the temperature, interfering with the critical fall, and it prolongs convalescence; exudation is revealed by flatter percussion note and lessened fremitus. Large effusions further seriously embarrass the respiration by pressure on the already crippled lung.

Whenever the crisis is unduly deferred or there exist signs of delayed resolution with anorexia and temperature, empyema should always be suspected and a needle introduced, for in the child bronchial breathing over the exudation is the rule, and if this fact be not remembered the effusion may escape recognition. Abscess, gangrene, and chronic caseous degeneration are rare possibilities. But, in general, the prognosis is excellent, the mortality being only about two per cent. of the cases, which fact further strongly distinguishes them from the next division of the subject.

Broncho-pneumonia is much the same insidious scourge of childhood in the winter months that the diarrheal diseases are in summer. Unlike the lobar form, it chooses as its victims not the strong and well nourished, but the great army of weak, enfeebled humanity who bear the traces of rhachitis, scrofula, and hereditary syphilis, and show the effects of vicious environment, improper feeding, and neglect. It decimates the foundling asylums. It seizes upon those reduced by all chronic or exhausting maladies, and ingrafts itself with especial predilection upon those suffering from measles, whooping-cough, and diphtheria. It is further distinguished from lobar pneumonia by its gradual onset, its prolonged and irregular course, and its slow defervescence by lysis.

Studied in the dead house, it is found to some degree in a much larger percentage of those who come to autopsy than would generally be supposed.* Almost every portion of the pulmonary structure may be involved, and with kaleidoscopic variety each new case presents a different picture from the last. Trachea, bronchi,

---

*Small areas are found, especially in the dependent portions of the lungs in a large proportion of all asthenic and marasmic cases.

alveoli, interstitial tissue, blood-vessels, pleura, and bronchial glands share in the process, some invariably, others but occasionally. The disease is primarily one of the bronchi, both large and small, the mucous membrane of which is congested, swollen, and covered with mucus, while the smaller bronchi are filled with frothy purulent secretion. The inflammatory infiltration is by no means limited to the mucous membrane, but involves chiefly the wall of the bronchus and extends by contiguity to the adjacent alveoli and the intra-alveolar connective tissue. The alveolar epithelium proliferates, and, together with white and red cells and fibrin from the swollen capillaries, is soon present in varying amount in the vesicular spaces. The course of a bronchus is then comparable to the path of a hot needle plunged into pulmonary tissues, searing the adjacent structures as well as its own immediate course.* All portions of the lungs are not affected to the same degree. The process may involve only the bronchus with its terminal cluster of alveoli and its contiguous vesicles, or the consolidation may extend in every direction. It may be limited to small disseminated areas, or it may involve the larger part of both lungs. Every variation between the extremes appears sooner or later upon the autopsy table.

But the consolidation does not always exist alone; three other factors—atelectasis, congestion, and emphysema—one or all, may unite to further disable the already crippled lung. The development of the atelectasis has been explained by Gardner † as follows: Inspiratory efforts draw tenacious secretion or pseudo-membrane deeper and deeper into the finer bronchi until it forms a valve-like plug at the entrance to the group of terminal alveoli. The residual air contained in the alveoli is then absorbed or else the strong expiratory efforts force it little by little past the obstruction, while the valve-like mass in the bronchus prevents the re-entrance of air during the weaker act of inspiration. Gradually the affected portion ceases to be aërated and the collapsed part is rendered useless and practically solid.

Again, I believe that certain hyperemic areas of the lung may

---

* Delafield. *Pathological Studies.*

† Pathol. Anat. of Bronchitis.

become so engorged and congested as to seriously interfere with the entrance of air into the alveoli and result in a condition which produces the same physical signs and the same evil effects as true consolidation. With the available respiratory surface much decreased, the efforts to expand the chest tend to overdistend the remaining alveoli, and this, aided by the expiratory pressure during cough, when the glottis is closed, may rapidly cause emphysematous dilatation of the vesicles, especially those along the anterior borders, where the lung, being thin, receives less support from the surrounding tissues.

If a lung from an extreme case, combining all these factors, is examined when first removed from the thorax, it presents an instructive study. The anterior borders of both lungs, and especially of the upper lobes, are smooth, tense, and prominent, rising sharply above the adjacent portions and having the pinkish-white color of distended lung. Closer inspection shows that the individual alveoli can be seen by the naked eye, while in places along the edges of the lobes larger distended alveolar spaces are readily apparent, due to the coalition of several vesicles. These emphysematous portions of the lung remain inflated because the natural contractile power of the lung is alone insufficient to drive the contained air through the accumulated secretion in the bronchi.

In close proximity to these emphysematous portions, or even extending into them or surrounded by them, may be seen depressed atelectatic areas, large or small, of a bluish or bluish-black color, where the occlusion of one or more terminal bronchi has caused the collapse of the area to which it gave access, while scattered over the rest of the lung are the true broncho-pneumonic areas, often upon the posterier surface and the lower dependent portions, but quite as often elsewhere, around the root of the lung, on the surface between the lobes, in the cardiac lingula, or in the right middle lobe. They are irregular patches of a dark-red color which are firm to the touch and have a coarse granular feel, rising above the level of the atelectatic patches but not so high as that of the emphysema.

Around some of these again, and contrasting both in color and resistance, may be seen violet-red areas of congestion, which are readily inflated; but the hyperemia which has given signs during life does not always persist after death. In some lungs we find small dark areas of lobular outline which are shown microscopically to consist of groups of alveoli whose cavities are filled with red blood-corpuscles only. These have, presumably, escaped by diapedesis from the engorged capillaries of the alveolar walls, which are seen to be distended with red cells. On section, the broncho-pneumonic areas are of a dark or light red, or grayish or yellowish pink, according to their age and the proportion of white cells in the exudate, and by pressure yellow purulent secretion may be made to well up from the opening of the smaller bronchi. Microscopically, the contents of the alveoli is similar to that in lobar pneumonia, though with less fibrin, but the essential difference lies in the infiltration of the walls of the bronchi and the inter-alveolar partitions, so that the disease is not only one of exudation but of proliferation.

The atelectatic portions, which are often superficial, vary much in the degree to which the atelectasis is developed. If this is extreme, they have the consistence of muscle, are flaccid, and do not crepitate on pressure, if they contain no air. Theoretically, also, they should for the same reason sink if placed in water, but often enough air remains in bits of tissue to buoy it up. Part of these lobules may still be inflated, although later inflammatory changes usually develop.

While I have given above the outline of what may be considered as strictly broncho-pneumonia, it should yet be remembered that there are cases of secondary pneumonia in which the areas of lobular involvement have no connection with the bronchi.

But it is not my desire to dwell upon the pathological or morphological aspect further than is necessary to throw light upon the course of the disease and its physical signs.

The symptoms and course of broncho-pneumonia depend so largely upon coexisting conditions, especially in those cases secondary to the infectious diseases, that it is more difficult than in lobar pneumonia to present a classical picture. In the primary cases there exists for a day or two a bronchitis which may be considered the first stage of the disease, and it is noticed that the cough becomes more frequent and hacking, the temperature rises, the face becomes anxious, the respirations more rapid, and the alæ nasi dilate with each inspiratory effort. When the process in the lung has advanced the child lies limply in its mother's arms, its face pale or livid, its skin hot and dry. In nursing or drinking it stops every few moments to struggle for breath. The lips are cracked, and the tip of the tongue often dry. If the chest be uncovered, the respiratory struggle becomes more apparent. Open mouth and dilating alæ nasi do not suffice to give it air. All the accessory muscles are called into play, but the consolidated and occluded portions of the lung cannot expand, a partial vacuum within the chest is formed, the episternal notch, supra-clavicular hollows, and epigastrium are sucked in with each breath, while deep furrows are formed in the intercostal spaces and the less resistant chest wall is drawn inward along the insertion of the rapidly contracting diaphragm. The respiratory center attempts to substitute speed for depth, and the respirations rapidly increase to seventy to eighty per minute. The pulse becomes 160 to 200. Each advance of the process in the lung, each transitory congestion, each area or collapse, adds to the intensity of the dyspnœa, and, unless relief comes, the respirations become more shallow, the pulse more rapid and feeble, the face assumes an ashen-gray color, and death ensues.

If, however, a happier issue obtains, the pulmonary obstruction decreases, respiration is accomplished with less effort, the sinking in of the chest ceases, the temperature declines from day to day, the pulse improves, the face assumes a better color, appetite returns, and convalescence is slowly established. It must not be supposed, however, that all cases follow these definite outlines or rise to this degree of severity. In no disease of childhood, perhaps, are there so many variations, such sudden changes, such prolonged suspense, such disappointing relapses.

In the above description I have purposely omitted all reference to the physical signs, because no greater error could be promulgated than an attempt to make them conform to any fixed standard. To them, or rather to the exact underlying condition of the lungs, pertains the greatly diversified interest of these cases. Upon the daily, almost hourly, changes in the lung depend the course and the prognosis, our hopes and our fears.

We have inflamed bronchi lined with swollen mucous membrane, filled with muco-purulent secretion, and giving rise to almost every variety of râle, sibilant and sonorous in the early stages, fine and coarse moist sounds in the later. These may be further modified as they come to the ear, depending upon the condition of the tissues through which they pass, and may have a ringing, metallic sound, or be harsh and crackling. Sudden atelectasis may give diminished or absent breathing where before there was a vesicular murmur. Subsequent changes may give rise to modified resonance or dull tympany. Existing atelectasis may disappear as suddenly as it came. The congestion which we found preceding or accompanying lobar pneumonia or existing by itself is even more important here. It may result in partial dullness, with diminished or broncho-vesicular respiration. It may change rapidly from place to place. It may be here to-day and gone to-morrow. Emphysema even helps forward the confusion and uncertainty, for it may be so near or so surround the patch of true consolidation that its hyper-resonance prevents the recognition of the dullness; and the exaggerated respiratory murmur may completely conceal the faint or distant bronchial breathing. Fibrinous pleurisy, although often present, seldom gives distinctive signs. Enlarged bronchial glands by pressure on a bronchus may cause diminished breathing.

We turn at last to the broncho-pneumonic consolidation for clear and unmistakable signs, but here again we are met with disappointment. We have seen already with what varying distribution and in what diverse forms these areas may appear. Sometimes in narrow zones about the bronchi, sometimes at the root of the lung or between the lobes; again in such small superficial areas as to furnish no clew to its existence, while only a moderate number of the consolidated areas give us definite and unequivocal signs.

You ask me for what purpose I have catalogued the exceptions and emphasized the difficulties. I answer, because in these very variations unmentioned by the usual text-books lie the characteristic features of the disease. Because the very things which would otherwise puzzle us and cause us to doubt the accuracy of our observations and even the evidences of our senses now point us unerringly to the diagnosis. Because, also, it shows us that we have of late years swung the pendulum too far in the direction of physical exploration and too far away from the careful semeiological inspection of the patient which Fothergill, as one of the last of the old school, tried so hard to impress upon the rising generation. In short, with certain conditions of the temperature and respiration, together with uncertain or shifting physical signs, we may feel as confident of our diagnosis of broncho-pneumonia as if we had discovered unmistakable signs in the lungs.

Let us see, however, whether it is not possible to bring order out of chaos and gain a clearer idea of the physical signs and their interpretations.

The broncho-pneumonic areas may vary from a slight exudation into a few alveoli immediately surrounding a bronchus and showing nothing on inspection, through the grades of larger visible zones about the bronchi, to the extensive consolidation of large portions of the lungs. The process is usually bilateral, though often patches large enough to be seen or to give physical signs are limited to one lung. When the patches of consolidation are small, or superficial, or disseminated, the difficulties are the greatest; when they are large and deep and the signs distinct, there is no difficulty in their recognition.

Exaggerated resonance may be elicited over the apices, especially anteriorly, where the emphysema is most commonly found. Normal resonance, with subcrepitant râles, usually indicates the presence of bronchitis only, but it is, of course, possible that areas of hepatization may be present, though so small or scattered as to add no signs. Much that is of value may be learned from close attention to the râles, for their coarseness or fineness corresponds more or less closely to the size of the bronchi involved, and their number to the intensity of the involvement. Where they are numerous and fine and the respiration embarrassed, we may suspect the presence of disseminated consolidation, although no other signs be present. Modified resonance indicates congestion or hepatization. Distinct dullness may be considered the exception rather than the rule.

A most important matter, however, is this fact: That both consolidation and fleeting hyperemia give identical signs, and it is necessary to have it firmly impressed upon our minds that only repeated examinations will determine the fixity or mobility of the signs, and so decide definitely with which process we have had to deal. Thus, if the signs of consolidation disappear in twenty-four to forty-eight hours, we know that we have dealt with an area of congestion; but if it persists, that it is hepatization. While, again, if it comes and goes, and finally returns to stay, we know that congestion, like a troop of horses, has swept hither and thither, preceding and concealing the main body of true consolidation, which was slowly advancing to occupy the territory.

There are frequent instances in which the atelectasis, congestion, and tenacious bronchial secretion tend to prevent the entrance of air into the disabled lung, there is diminished breathing over the affected area, and the râles conceal its faint bronchial character. In these cases the compensatory efforts of the other lung are attended with loud consonant râles and harsh exaggerated respiration. Here the temptation to the careless or superficial examiner is always to locate the lesion where there is the most noise. No portion of the chest should be omitted in the examination, for the sides and the axillæ, the right middle lobe, and cardiac lingula are as often involved as the apices or the bases.

The temperature charts of broncho-pneumonia exhibit no characteristic course. The febrile period, corresponding to the active process in the lung, is of longer duration than in lobar pneumonia; it declines by lysis, and it shows greater daily variations. Although several charts placed side by side show no special uniformity, there are several general rules which have been deduced after careful comparison of the charts with the record of the physical signs. The extensions and recessions of the inflammatory process in the lung are thus seen to be registered, to some extent, upon the temperature chart. Thus bronchitis of the large and medium-sized tubes causes moderate elevation of temperature with partial morning remissions. When the finer bronchi becomes involved the elevation is greater, even to 104° or more. Congestion of fresh portions of the lung shows itself by sudden, sharp exacerbations of temperature as it develops, and equally sharp falls when it disappears, the degree depending upon the amount of lung involved and the intensity of the process it accompanies. Hepatization reveals itself only by its tendency to maintain the curve at a more uniform level.* Thus, as these three factors—bronchitis, congestion and hepatization—ingraft themselves with varying intensity upon different portions of the lung, they are reflected in an infinite variety of combinations upon the record of the temperature.

The favorable cases, from a prognostic standpoint, are those which run their course between 101° to 104·5° F. Patients with an excessively high temperature usually die, as do those in whom it never rises above 101° F., or is even subnormal throughout. In these latter cases the low temperature seems to be an index of the feebleness and low vitality of the child.

The mortality has been estimated to vary from thirty to seventy per cent., according to the age of the patient and the circumstances under which the disease develops. The younger the child, the greater the danger. Among the primary cases the previous condition and constitution of the patient are important factors. Among the secondary cases the severity and nature of the primary affection must be considered. Cases complicating measles and pertussis give us our highest mortality. Institution children succumb readily; the children of the well-to-do fare the best. The physical signs are of but moderate assistance, remembering that extensive lesions may be so situated as to give no signs, and that extensive signs may represent superficial and transitory lesions.

It is, after all, to the rational signs that we must turn for our prognostic view of the case. If the respiration becomes superficial, or the cough ceases, or the skin becomes ashen gray, death will ensue if relief is not quickly given. The influence of low and persistently high temperatures we have already seen. Moderate increase of the consolidation is not as bad as extensive congestion or atelectasis. A sudden exacerbation of the catarrhal bronchitis may cause death in a few hours from suffocation.

Favorable cases defervesce in from seven to twenty-one days, though cases may run on for weeks, and the chances of recovery in these prolonged cases steadily decrease with the lapse of time. Reso-

*Cadet de Gassicourt. *Maladies de l'enfance*, Paris, 1886.

lution requires seven to fourteen days for its completion, for here there is not only the exudation into the alveoli, but the infiltration of the inter-alveolar connective tissue and the walls of the bronchi, which must be absorbed. Occasionally it extends over a period of eighteen to twenty-eight days. In some instances resolution does not occur for many months and then gradually takes place. In others the walls of the air spaces become thickened, the alveoli themselves may even be filled with organized tissue, and a chronic fibroid condition results, with contraction of the lung and chest and subsequent dilatation of the bronchi. If the area involved be large, these latter cases, with rare exceptions, do not survive long, but die of intercurrent disease or develop tuberculosis.

Very little time is left us for the consideration of the treatment. As the indications for both forms are similar, they can be spoken of together. Many years ago pneumonia was treated by bloodletting, calomel, and especially on the continent by large doses of the emetic drugs—ipecac and antimony—until Barthez* stated that in his opinion meddlesome medicine prolonged rather than shortened the course of the disease, if it did not, indeed, largely increase the mortality. A more intimate knowledge of the affection now directs our more intelligent therapeutic efforts. The propriety of prophylaxis needs but to be mentioned. Prompt attention to all minor inflammatory conditions of the air passages is to be insisted upon and as quickly dismissed.

What, then, is the *rationale* of the modern treatment of the disease itself? As pneumonia is a self-limiting malady, we adopt the expectant plan of treatment. Temperature, heart action, and respiratory function give us our chief indications of interference. Although the deleterious influence of prolonged high temperature upon the tissues is well known, cases vary greatly in the degree to which they show these evil effects. It is rather the effect of the temperature upon the heart, the brain, and the nervous system which we should consider rather than its height in degrees. Each case must be judged by itself. If it is producing deleterious effects, it should be promptly combated, and to this end the bath or the wet pack are undoubtedly the best measures, for they do not act alone by the ab-

*_Mém. de l'Acad. de Médecine_, Paris, 1862.

straction of heat, but are stimulant as well as antipyretic. Not only does the bath produce a subjective feeling of comfort in the patient, but it stimulates the cutaneous vessels, restoring their tone and relieving the overburdened heart; it also calms the central nervous system, removes delirium, favors sleep, and restores the nervous control over the heart and the functions of the other organs.

I have purposely avoided the use of the word "cold" in referring to both baths and packs, for there are conditions where the rectal temperature registers 105° to 106° F., but the surface of the trunk, and especially of the extremities, is cool and the shock of a cold bath would be a source of great danger. The temperature of pneumonia yields more readily to the water treatment than that of typhoid fever, and a bath of 95° F., cooled slowly by the addition of cold water to 85°, or in sthenic cases to 75°, and continued for ten to fifteen minutes, is sufficient to produce a reduction of 2° to 3° in the temperature. Exactness is as necessary here as elsewhere, and the watch and the bath thermometer are as indispensable to the bath as the scale and the graduate to intelligent medication. During the bath friction should be applied by the palm of the hand to the surface of the body, and on removal the patient should be wrapped in a blanket, or sheet and blanket, and allowed to rest for a time undisturbed. Stimulants may be necessary both before and after the bath.

Still better and far more convenient is the wet pack, the child being wrapped up to the neck in sheets wet in water at 75° to 85° F., and covered with a blanket. These may be changed several times at intervals of ten minutes, the last being allowed to remain for half an hour. Twice or three times a day will usually be sufficient. The other antipyretics are not to be entirely discarded, but, if used, their depressant effects must be remembered and they should be guarded by the administration of stimulants.

More important, however, than the temperature is the respiratory function. The danger to adult and child from pneumonia rests upon entirely different bases. In the adult the respiratory muscles are well developed and the strain comes on the relatively weak right heart and the danger is cardiac failure. In the child, however, the two sides of the heart have more nearly the same power, but the thorax is yielding and the respiratory muscles have not reached their full development. Accumulation of secretion in the bronchi, congestion, and atelectasis are therefore especially to be feared. Emetics to empty the bronchi are but rarely indicated, because of their depressant action. This indication is best met by sustaining the strength by proper feeding and the administration of stimulants, digitalis, strychnine, alcohol, or carbonate of ammonia. Congestion should be avoided or combated by counter-irritation to the chest, by mustard pastes, mustard baths, the application of camphorated oil or turpentine, and olive oil, equal parts, and the use of the oil-silk jacket. In some cases dry cups are of great service.

I have only been able to allude hurriedly to these few important factors in the treatment. In conclusion, I desire to emphasize—

1. The frequency of true lobar pneumonia in young children.

2. The misleading character of the cerebral and gastric types of lobar pneumonia.

3. The fact that pneumonia is often overlooked, and the necessity of repeated examinations of the chest in all sick children, especially of those with persistently high temperature.

4. The importance of examining all portions of the chest.

5. The very variable distribution and extent of the broncho-pneumonia lesions.

6. The importance, both from a morphological and from a clinical standpoint, of emphysema, atelectasis, congestion, and intra-alveolar hemorrhage.

7. The frequent discrepancy between the physical signs and the symptoms of broncho-pneumonia.

8. The falsity of the impression that distinct signs of consolidation are necessary to the diagnosis of broncho-pneumonia.—*N. Y. Med. Jour.*

19 *West Forty-sixth Street.*

